# Targeting of the Tumor Necrosis Factor Receptor Superfamily for Cancer Immunotherapy

**DOI:** 10.1155/2013/371854

**Published:** 2013-06-11

**Authors:** Edwin Bremer

**Affiliations:** Department of Surgery, Translational Surgical Oncology, University Medical Center Groningen, University of Groningen, 9713GZ Groningen, The Netherlands

## Abstract

The tumor necrosis factor (TNF) ligand and cognate TNF receptor superfamilies constitute an important regulatory axis that is pivotal for immune homeostasis and correct execution of immune responses. TNF ligands and receptors are involved in diverse biological processes ranging from the selective induction of cell death in potentially dangerous and superfluous cells to providing costimulatory signals that help mount an effective immune response. This diverse and important regulatory role in immunity has sparked great interest in the development of TNFL/TNFR-targeted cancer immunotherapeutics. In this review, I will discuss the biology of the most prominent proapoptotic and co-stimulatory TNF ligands and review their current status in cancer immunotherapy.

## 1. Introduction

The tumor necrosis factor (TNF) superfamily is comprised of 27 ligands that all share the hallmark extracellular TNF homology domain (THD) [1]. This THD triggers formation of non-covalent homotrimers. TNF ligands are typically expressed as type II transmembrane proteins,but in most ligands the extracellular domain can be subject to proteolytic processing into a soluble ligand. TNF ligands exert their biological function by binding to and activation of members of the TNF receptor (TNFR) superfamily. These TNFRs are typically expressed as trimeric type I transmembrane proteins and contain one to six cysteine-rich domains (CRDs) in their extracellular domain [2].

The TNF ligand superfamily has diverse functions in the immune system, one of which is the induction of apoptotic cell death in target cells. This function is performed by a family subgroup coined the Death Inducing Ligands, comprising the archetypal member TNF, FasL, and TRAIL. These Death Inducing Ligands bind to and activate cognate members of a TNFR subgroup termed the Death Receptors (DRs). DRs are characterized by the hallmark intracellular Death Domain (DD) that transmits the apoptotic signal. In general, ligand/receptor interaction induces formation of a Death Inducing Signaling Complex (DISC) to the cytoplasmic DD [3]. This DISC comprises the adaptor protein Fas-associated death domain (FADD) and an inactive proform of the cysteine protease procaspase-8. In addition to procaspase-8, the inhibitory caspase-8 homologue cFLIP can be recruited to this complex [4]. Within the DISC, caspase-8 is auto-proteolytically processed via proximity-induced activation [5], whereupon a catalytic caspase-mediated pathway of apoptosis ensures execution of apoptotic cell death. All of these three proapoptotic TNF ligands hold considerable interest for tumoricidal cancer therapy [6].

A second important function of the TNF superfamily is the provision of co-stimulatory signals at distinct stages of an immune response [7]. Such co-stimulatory signaling is initiated upon TNFL/TNFR interaction and subsequent recruitment of members of the adaptor protein family of TNF receptor associated factor (TRAFs) [8]. The TRAF family consists of 6 members and is characterized by a highly conserved C-terminal domain that is responsible for trimer formation and interaction with the TNF receptors. The N-terminal domain is less conserved and is responsible for downstream proinflammatory and prosurvival signal transduction [9]. Typical signaling pathways activated by TRAFs are NF*κ*B, PI3K, and PKB. Various co-stimulatory TNFL/TNFR pairs, including CD40L/CD40, CD70/CD27, 4-1BBL/4-1BB, and OX40L/OX40, have gained prominence as possible targets for cancer immunotherapy, in particular with the aim of induction or (re)activation of antitumor T-cell immunity.

**Figure 1:**
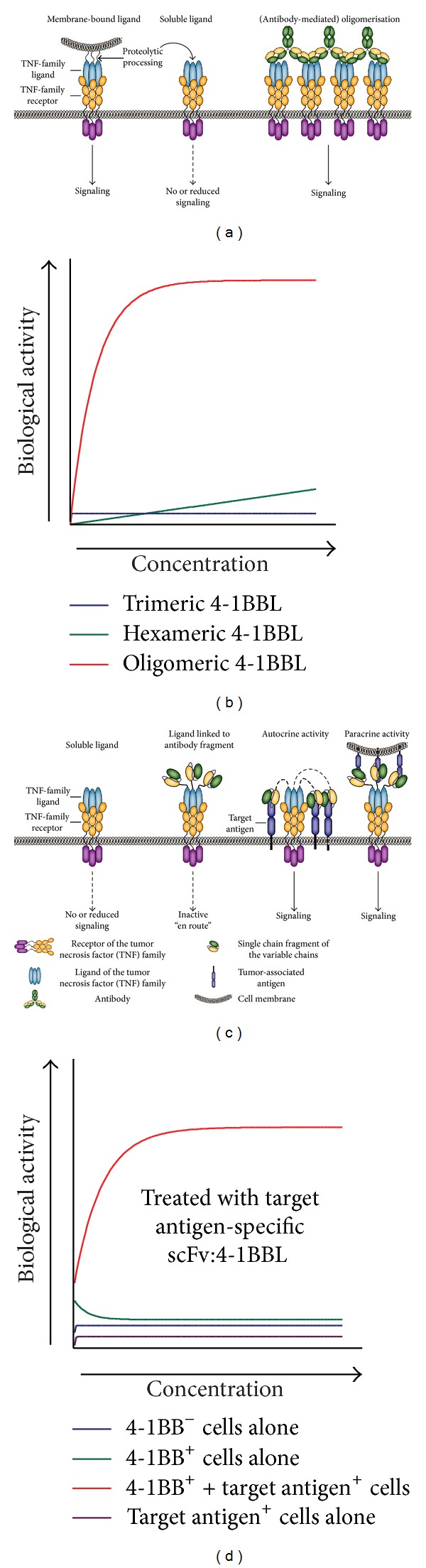
TNFL/TNFR signaling characteristics. (a) TNF-ligands are typically produced as type II transmembrane proteins, but the extracellular domain of most of these ligands can also be proteolytically cleaved by proteases, such as ADAM-17 (also known as TACE) [10], into a soluble form. Typically, the soluble ligand retains binding activity but has lost some or all receptor-activating activity. This activity can be restored by secondary cross-linking. (b) Signaling requirements of 4-1BB-signaling by s4-1BBL. (c) The cross-linking requirement of sTNF ligands makes their inclusion into an antibody fragment approach attractive. In brief, such a TNFL-fusion protein comprises a scFv antibody fragment genetically fused to the TNFL. This scFv:TNFL fusion protein is essentially inactive en route. However, upon target binding of the scFv antibody fragment domain the soluble ligand is converted into a signaling competent membrane-like ligand. (d). Illustration of target cell-restricted activation by scFv:4-1BBL.

As briefly mentioned earlier, a prominent feature of most TNF ligands is their proteolytic processing into soluble trimeric ligands (Figure 1(a)). Many of these soluble TNF ligands have a significantly reduced signaling activity compared to their transmembrane counterparts. Whereas the soluble trimeric ligand typically still binds the receptor, it requires secondary cross-linking to achieve TNFR activation reminiscent of its transmembrane TNF ligand counterpart as illustrated in Figure 1(b) for 4-1BBL. This feature of the TNF superfamily has formed the basis for their incorporation into antibody-based targeted therapies (see Figure 1(c)). In brief, a TNF ligand is genetically fused to a tumor specific antibody fragment, yielding a soluble targeted TNF ligand that is essentially inactive “en route.” Upon antibody fragment binding to the target cells, this ligand is converted into a membrane-associated and fully signaling competent form of the TNF ligand as illustrated in Figure 1(d) for tumor targeted 4-1BBL.

In this review, I will first briefly describe the biology and then provide an overview of the state-of-the-art of therapeutic exploitation of the proapoptotic ligands TNF, FasL and TRAIL. Second, I will similarly review several prominent immune co-stimulatory TNF ligands, in particular CD40L, CD27L, 4-1BBL, and OX40L. For each of these ligands, I will further detail the rationale for their inclusion in antibody-mediated targeting to achieve tumor-selective activity and reduced toxicity towards normal cells.

## 2. Tumor Necrosis Factor (TNF)

### 2.1. TNF Biology

TNF is the archetype superfamily member and is mainly produced not only by macrophages, but also by a broad variety of other cells, including lymphoid cells, mast cells, endothelial cells, fibroblasts, and neuronal tissue. TNF-alpha interacts with two receptors, namely TNF receptor 1 (TNFR1) and TNF receptor 2 (TNFR2). TNFR1 is constitutively expressed in most tissues and contains a cytoplasmic DD and as such is capable of transmitting TNF-mediated proapoptotic signaling. TNFR2 lacks a cytoplasmic DD and is expressed predominantly on immune cells and endothelial cells.

Despite the fact that TNFR1 contains the hallmark DD required for induction of apoptosis, the main signaling outcome of TNF/TNFR1 interaction is not apoptotic cell death. Instead, TNF-induced TNFR1 signaling typically activates classical nuclear factor kappa B (NF*κ*B) proinflammatory signaling [11]. In brief, binding of TNF to TNFR1 recruits the DD-containing adaptor molecules TRADD and RIP1. TNFR1-bound TRADD subsequently recruits TRAF2 and cIAP-1 and -2, whereupon NF*κ*B as well as c-Jun N-terminal kinase (JNK) signaling is induced [12, 13]. 

Upon receptor internalization this primary complex dissociates from TNFR1, after which a secondary complex comprising FADD, procaspase-8 and cFLIP can be formed [11]. If cellular cIAP and cFLIP levels are limited, this secondary complex subsequently induces apoptosis via caspase-8. Notably, binding of membrane TNF to TNFR2 modulates TNFR1 signaling via proteasomal degradation of TRAF2 and cIAP. As a consequence, TNFR2 can tip the balance from inflammatory to apoptotic TNFR1 signaling (reviewed in [14]).

TNF is a major proinflammatory mediator of the innate immune system and can exert a large spectrum of bioactivities. Indeed, TNF modulates a host of (patho)physiological processes and is, for instance a critical mediator of shock and involved in both tissue regeneration/expansion and destruction [14]. As described earlier, the formation of the second signaling complex also enables TNF to trigger apoptotic cell death in certain circumstances. For instance, TNF proved to have potent tumoricidal activity *in vitro* and in mouse models in initial studies [15, 16], a finding that sparked interest in the development of TNF for cancer therapy.

### 2.2. Triggering TNF/TNFR Signaling for Cancer Therapy

**Figure 2:**
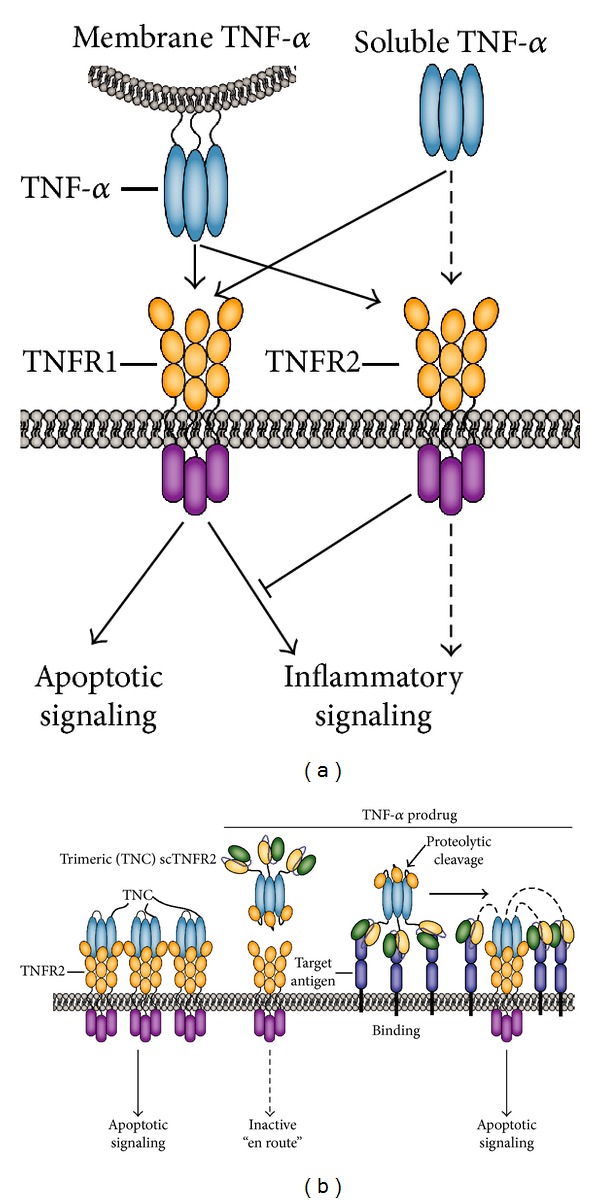
TNF/TNFR signaling and TNFR-targeted therapeutics. (a) TNFR1 and TNFR2 are effectively activated by membrane TNF, but sTNF can only trigger TNFR1-signaling. (b) TNFR-targeted drugs include a stabilized TNFR2-selective scTNF that may help to induce TNFR1 proapoptotic signaling, as well as targeted strategies such as scFv:sTNFL, and scFv:sTNF-TNFR1 prodrug constructs. The latter only become activated after target antigen-selective binding and subsequent cleavage of the TNFR1 inhibitory domain by tumor-overexpressed proteases.

Like most family members TNF is a transmembrane protein [17], but its extracellular domain can be proteolytically cleaved into a soluble form (sTNF) [10]. Of note, TNFR1-mediated downstream signaling is induced with similar efficacy by membrane TNF and sTNF (Figure 2(a)). In contrast, TNFR2 is poorly activated by sTNF and requires membrane TNF for efficient signaling [18]. In preclinical studies, recombinant sTNF displayed potent tumoricidal activity [16, 19]. Unfortunately, systemic administration of recombinant sTNF only yielded minimal clinical activity in phase I clinical trials [20, 21] and was, moreover, associated with severe dose-limiting toxicity already at low doses. These initial findings clearly negated the use of sTNF as a systemic cancer therapeutic modality. Nevertheless, locoregional use of soluble TNF in combination with the chemotherapeutic drug melphalan yields impressive clinical responses in isolated limb and isolated liver perfusion [22, 23] and has become part of clinical practice. In these locoregional applications, sTNF is infused at over 50 times the maximal tolerated dose (MTD) as identified during systemic sTNF therapy. This high dose of TNF triggers endothelial cell apoptosis and subsequent destruction of the tumor vasculature, whereas normal blood vasculature is not affected. Consequently, tumor penetration of melphalan is enhanced. 

Of note, the requirement for high concentrations of sTNF in isolated liver or limb perfusion indicates that in addition to TNFR1, TNFR2 signaling is required to sensitize tumor vasculature to apoptotic TNFR1 signaling. In this respect, the combined use of a low dose of sTNF with a TNFR2-selective TNF variant may optimize therapeutic effects on tumor vasculature and minimize toxicity. Of interest in this respect is a newly reported soluble TNFR2 agonist (TNC-scTNFR2), in which the trimerization domain of tenascin C (TNC) was fused to a TNFR2-selective single-chain TNF molecule comprised of three TNF domains connected by short peptide linkers (Figure 2(b)) [24]. TNC-scTNFR2 specifically activated TNFR2 and, importantly, possessed membrane-TNF like activity towards TNFR2.

### 2.3. Targeted TNF-Based Cancer Immunotherapy

The major TNF receptor, TNFR1, is ubiquitously expressed on normal cells, as a consequence of which soluble TNF has no or only limited tumor binding selectivity and considerable toxicity. Further, soluble TNF (sTNF) is practically incapable of triggering TNFR2 signaling and can only efficiently activate TNFR1, whereas TNFR2 signaling positively affects the efficacy of TNFR1 apoptotic signaling. All of these features clearly position TNF for inclusion in tumor targeting strategies, a strategy that has been pursued by various groups using scFv:TNF-based fusion proteins (Figure 2(b)). Such scFv:TNF fusion protein will selectively bind to the corresponding target antigen via the antibody fragment. This high affinity interaction will ensure target cell accretion and, importantly, converts the sTNF domain into membrane-like TNF. Consequently, target cell-bound scFv:TNF can efficiently activate TNFR2 and thereby sensitize cancer cells to induction of TNFR1 apoptotic signaling. Proof of concept for antibody-mediated delivery of sTNF has been generated for stromal (FAP), endothelial (integrins), and cellular targets (e.g., EGFR and Her2) (reviewed in [25]), with very favorable tumor-to-blood ratios 2-3 days after injection and potent tumoricidal activity at doses below the Maximum Tolerated Dose (MTD) of sTNF in preclinical models [26–28]. Nevertheless, soluble TNF remains highly active towards TNFR1 expressed on normal cells in the scFv-TNF format. Further, target antigens used to deliver TNF in these scFv-TNF fusion proteins are often not tumor specific but tumor associated or overexpressed, such as the Epidermal Growth Factor Receptor (EGFR) [29] or Her2 [30]. Consequently, ubiquitous TNFR1 activation or off-target activation of scFv-TNF on normal target antigen-positive cells may occur upon infusion of scFv-TNF fusion proteins with the ensuing potential for toxicity such as those seen for sTNF [27, 31]. 

To further decrease the potential for toxicity issues, strategies that unleash the full tumoricidal potential of sTNF only at the intended site of action have been developed. Various recent advances have indeed made significant headway towards achieving such tumor-selective activation of sTNF. Firstly, sTNF was genetically fused to a fibroblast-activation-protein (FAP) specific antibody fragment that additionally contained the immunoglobulin dimerization domain [32, 33]. This dimerization domain ensured that the scFvFAP-TNF fusion protein formed relatively inactive dimers in solution, leading to a 10–20-fold reduction in bioactivity compared to sTNF. However, upon FAP-selective binding the scFvFAP-TNF fusion protein regained potent TNFR-mediated cytotoxicity. Alternatively, antibody fragment-TNF fusion proteins have been C-terminally fused with the ligand-binding domain of TNFR1. This TNFR1 domain binds and shields TNF “en route,” yielding an inactive prodrug (Figure 2(b)). In this prodrug a special linker, designed to be sensitive to tumor-overexpressed proteases, was incorporated in between the TNF and TNFR1 domain. Consequently, the inhibitory TNFR1 fragment present in the prodrug will be cleaved by tumor overexpressed proteases and shed once at the site of the tumor. Proof of concept for this prodrug approach has been generated for the tumor stroma marker FAP with urokinase plasminogen activator (uPa) and matrix metalloproteinase-2 (MMP2) mediated cleavage of the TNFR1 domain [34, 35].

### 2.4. Perspectives for the Use of sTNF in Cancer Immunotherapy

The potential for systemic application of sTNF in cancer patients critically depends on the tumor-selectivity of the recombinant sTNF-based drug. The above-described tumor-targeted antibody fragment-targeted TNF fusion proteins have these properties and should have a significantly improved toxicity profile while retaining potent tumoricidal activity. Thus, these targeting concepts generate hope for the applicability of this cytokine for systemic cancer therapy. However, it remains to be shown in *in vivo* follow-up studies that such more advanced strategies retain effectiveness and can be sufficiently cleaved and activated at the site of the tumor. In patients, this will to a great deal depend on the tumor microenvironmental concentration of active proteases, a characteristic that will have to be determined to identify patients likely to benefit from such therapeutics. Further, TNFR2 selective variants such as discussed earlier [24] offer the possibility of combining low dose sTNF and TNFR2 selective variants possibly in sequential treatment schedules to generate synergistic apoptotic antitumor activity with minimal side effects.

Of note, endogenous tumor-produced TNF can have a remarkable protumorigenic activity [19], for instance by inducing expression of interleukin 13 receptor alpha 2 (IL13R*α*2) on tumor resident monocytic cells and the subsequent production of the immunosuppressive cytokine TGF-beta [36]. Importantly, antagonistic antiTNF antibody treatment blocked IL13R*α*2 expression and restored active antitumor immune activity in colorectal, fibrosarcoma, and pancreatic murine models [36, 37]. Thus, for recombinant sTNF derivatives the intratumoral TNFR cross-linking characteristics as well as intratumor concentration will be of critical importance to ensure tumoricidal activity in the absence of any low-dose and sub-optimal signaling mediated prosurvival signaling. Of note, blocking such prosurvival signaling may be a target for combinatorial design of TNF-based drugs with, for example, NF*κ*B inhibitors, such as parthenolide, or clinical grade proteasome inhibitors such as bortezomib.

## 3. Fas-Ligand (FasL)

### 3.1. FasL Biology

FasL (also known as CD95L) is a type II transmembrane protein expressed on immune effector cell such as T-cells. The main receptor for the ligand of FasL is the type I transmembrane receptor Fas (CD95) [38, 39], which is expressed on a variety of normal human cells as a homotrimer [40–42]. Binding of FasL to Fas triggers oligomerization of Fas and initiates apoptotic signaling by DISC formation at the intracellular DD [3]. Of note, nonapoptotic NF*κ*B-mediated proinflammatory signaling can also be induced by FasL [43, 44], although this signal is usually overruled by the apoptotic signaling pathway. However, in cells with high endogenous levels of apoptosis inhibitors, such as Bcl-2 or Bcl-xL-expressing cells, NF*κ*B-signaling by Fas can dominate. FasL can further interact with decoy receptor 3 (DcR3) [45], a soluble receptor that competitively inhibits FasL/Fas-mediated signaling. Indeed, high circulating levels of DcR3 have been shown to protect against FasL signaling in autoimmunity, for instance, by protection of synovial fibroblasts in rheumatoid arthritis [46]. 

**Figure 3:**
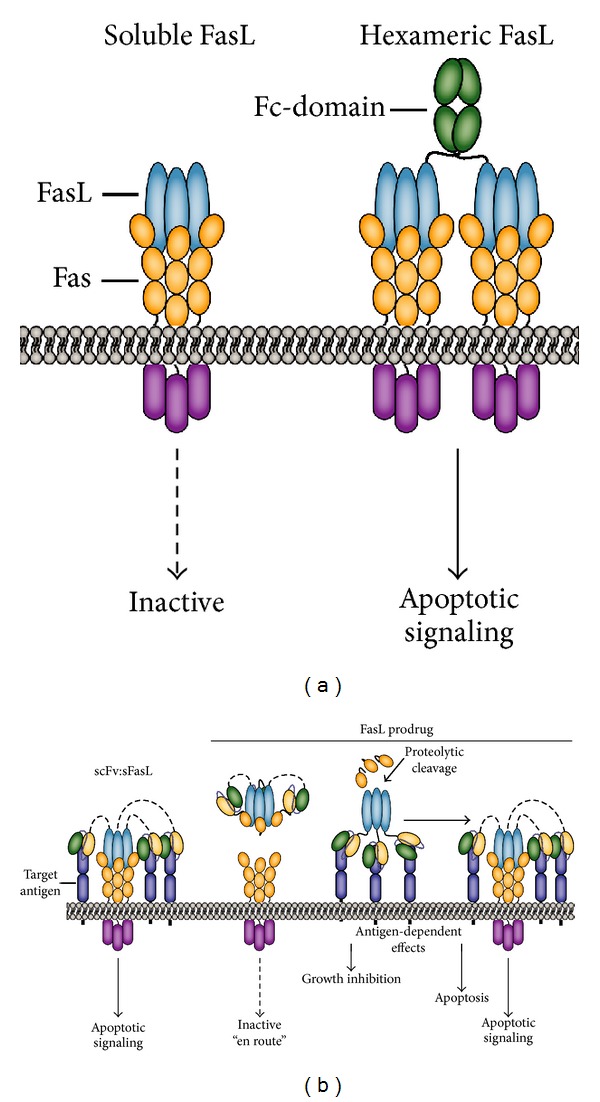
Design of FasL/Fas-based cancer therapeutics. (a) Soluble homotrimeric FasL is essentially incapable of activating Fas-apoptotic signaling. However, hexamerized recombinant forms of sFasL have Fas-activating capacity analogous to membrane-expressed FasL. (b) The inactivity of homotrimeric sFasL has been exploited in scFv:FasL fusion proteins, by which the full apoptotic potential of FasL/Fas signaling is unleashed only upon target antigen-selective binding. To further increase the safety of FasL-based therapeutics, a FasL-based prodrug strategy analogous to TNF has been designed and evaluated.

Next to the granzyme/perforin cytolytic pathway, the FasL/Fas system represents a major cytolytic effector route of CD8+ T-cells pivotal for elimination of target cells [47, 48]. Further, Fas-apoptotic signaling is also crucial for the reciprocal elimination of activated T cells during the resolution phase of a T-cell immune response [49, 50]. Of note, like most TNF ligands the extracellular domain of transmembrane FasL is subject to proteolytic cleavage, which generates soluble homotrimeric FasL (sFasL). Elevated serum levels of sFasL have been documented in pathological conditions among others in lymphoma patients [51] and synovial fluid of rheumatoid arthritis patients [52]. Importantly, processed sFasL is approximately 1000-fold less effective in inducing Fas signaling than transmembrane FasL (Figure 3(a)) [53, 54]. Thus, endogenous sFasL likely competitively inhibits the activity of immune effector cell-expressed full length FasL. 

### 3.2. Triggering FasL/Fas Signaling for Cancer Therapy

The prominent role of FasL/Fas signaling as cytolytic pathway in T-cell immunity sparked great interest in its use in cancer therapy. Indeed, agonistic human antiFas antibody triggered potent cytotoxicity in human xenografted tumors in murine models [38]. Unfortunately, upon further evaluation of Fas-based therapy in syngeneic murine tumor models and murine Fas-specific antibodies it became apparent that ubiquitous Fas activation is associated with severe lethal hepatotoxicity within 6 h of injection [55]. These early findings negated the systemic use of Fas agonists for cancer therapy. Nevertheless, compartmentalized activation of Fas signaling proved to be effective in murine models without serious adverse toxicity. For instance, intraperitoneal administration of sFasL efficiently eliminated murine lymphoma cells in the absence of toxicity [56]. Together, these data indicate that compartmentalized use of ubiquitous Fas-agonists or the design of more target-selective agonists holds considerable therapeutic potential.

In recent years, efforts have focused on the design of such more selective tumoricidal Fas agonists. As described earlier homotrimeric sFasL is inactive/poorly active even at very high concentrations. However, hexameric recombinant forms of sFasL, such as Fc-FasL or mega-FasL, were fully capable of activating Fas-apoptotic signaling in malignant hematopoietic cells while retaining an acceptable toxicity profile in mice (Figure 3(a)) [57, 58]. More recently, a polymeric dodecamer FasL chimera was described in which FasL was fused to the homotypic aggregation domain of Leukemia Inhibitory Factor receptor gp1909. This multimeric FasL chimera also proved highly active *in vitro*, possessed tumoricidal activity *in vivo*, and had no liver toxicity at tumoricidal concentrations [59]. Thus, it is possible to design sFasL variants that possess a clear therapeutic window, at least in murine model systems.

### 3.3. Targeted FasL-Based Cancer Immunotherapy

As described above, homotrimeric sFasL is inactive but can be reactivated by virtue of oligomerization. Similarly, immobilization of sFasL on extracellular matrix components restored membrane FasL-like apoptotic activity [60]. These characteristics establish sFasL as a prime target for inclusion in antibody-targeted scFv-based therapy (Figure 3(b)). In an scFv:sFasL-based fusion protein the antibody fragment will ensure high affinity and selective binding to the predefined target. Consequently, the inactive sFasL domain is converted into a membrane-like FasL that can activate Fas-apoptotic signaling. Proof of concept for this strategy was obtained for several target antigens, including the tumor stroma marker FAP [61], the T-cell surface markers CD7 [62], and the B-cell surface marker CD20 [63]. Specifically, FAP-selective binding to FAP-positive HT1080 cells induced Fas apoptotic signaling at a 1000-fold lower ED50 compared to corresponding parental FAP-negative HT1080 cells [61]. Further, CD7-specific binding of scFvCD7:sFasL triggered apoptotic elimination of CD7-positive T-cell leukemic cells but lacked activity on CD7-negative cells [62]. Of note, scFvCD7:FasL proved inactive towards normal CD7-positive cells, including resting T-cell and NK-cells. In contrast, activated T-cells were sensitive to apoptotic elimination by scFvCD7:FasL, probably due to the intrinsic increase in sensitivity to AICD occurring in T-cells at later activation stages. In actual fact, this T-cell restricted activity profile also enabled the selective elimination of pathogenic synovial fluid T-cells [64].

Interestingly, inclusion of a rituximab-derived antiCD20 antibody fragment in an scFv:sFasL fusion protein revealed a dual tumoricidal activity [63]. In brief, CD20-selective binding of scFvRit:sFasL induced CD20-apoptotic signaling, a finding corresponding with published reports that rituximab can activate apoptosis by cross-linking of CD20 [65]. Second, CD20-immobilized scFvRit:sFasL efficiently triggered Fas-apoptotic signaling. Of note, dual treatment of malignant B-cells with rituximab and agonistic antiFas antibody also triggers synergistic apoptotic cell death [66, 67]. However, in a side-by-side comparison scFvRit:sFasL proved superior to cotreatment with rituximab and sFasL or antiFas antibody.

### 3.4. Perspectives for FasL/Fas-Based Cancer Immunotherapy

To therapeutically exploit Fas-apoptotic signalling it is imperative to prevent ubiquitous activation of this receptor, particularly in the liver. The use of homotrimeric scFv:sFasL fusion proteins appear to fulfil this requirement, with as >1000-fold increase in agonistic activity upon target antigen-selective binding. However, the safety of this strategy still depends on the expression pattern of the targeted antigen. Indeed, most established target antigens in antibody-based therapy are not tumor specific but tumor associated and tumor-overexpressed. To further maximize tumor-restricted activation a pretargeting approach may be developed. For instance, sFasL can be fused to a fluorescein- (FITC)-specific scFv. Cancer lesions can be first targeted using an FITC-labelled antitumor antibody. Once the optimal tumor-to-tissue ratio has been achieved for this antibody, scFv-FITC:sFasL can be infused to enable FITC-specific and cancer lesion restricted accretion of scFv-FITC:sFasL. An analogous approach has been used for the selective elimination of Fas-scFv-FITC transduced cells as a conditional death switch [68]. 

Alternatively, a prodrug scFv:FasL-based strategy analogous to that reported for scFv:TNF has been pursued to ensure tumor-restricted FasL-induced signaling [69]. In this case, the receptor Fas and a TNC trimerization domain was N-terminally fused via a protease sensitive linker to the scFv:FasL construct. In this prodrug format, scFv:FasL fusion remained inactive en route. However, upon target antigen binding, tumor-overexpressed proteases cleaved the inhibitory Fas fragment, whereby FasL-apoptotic signalling was unmasked. Proof of concept *in vitro* as well as *in vivo* for such tumor selective activation was reported for the stromal marker FAP and the proteases uPA and MMP2 [69]. 

## 4. TNF-Related Apoptosis Inducing Ligand (TRAIL)

### 4.1. TRAIL Biology

TRAIL is expressed on various immune effector cells and binds to 4 surface receptors of the TNFR superfamily, TRAIL-R1 (DR4), TRAIL-R2 (DR5), TRAIL-R3 (DcR1), and TRAIL-R4 (DcR2) (reviewed in [70]). Of these four, TRAIL-R1 and TRAIL-R2 contain the characteristic DD required for induction of apoptosis. TRAIL-R3 is a phospholipid-anchored receptor, whereas TRAIL-R4 contains a truncated intracellular domain. Consequently, neither TRAIL-R3 nor TRAIL-R4 are capable of apoptotic signaling and are thought to have a decoy function. Of note, evidence for formation of heteromeric TRAIL-receptors comprised of TRAIL-R2 and TRAIL-R4 highlights an added layer of complexity to TRAIL/TRAIL-receptor mediated signaling that is yet to be fully elucidated [71, 72]. TRAIL can further bind to osteoprotegerin, whereby it may regulate vascular biology [73] and bone turnover [74].

TRAIL is an effector molecule involved in antiviral and antitumor immune responses, particularly in tumor immune surveillance by liver NK-cells, as TRAIL^−/−^ mice develop increased liver metastases [75]. Expression of TRAIL is induced by interferons on CD4+ and CD8+ T-cells [76], in virally infected cells [77], and on multiple myeloma cells [78]. Further, TRAIL was shown to be required for graft versus tumor activity upon allogeneic hematopoietic stem cell transplantation [79].

Apoptotic signaling by TRAIL via TRAIL-R1/-2 is similar to Fas-induced apoptotic signaling, with formation of the DISC, processing of caspase-8, and subsequent downstream caspase-mediated apoptotic signaling. Of note, in the presence of high levels of endogenous apoptosis inhibitors, such as cFLIP, TRAIL can also trigger proinflammatory NF*κ*B-signaling. Such proinflammatory signaling can actually promote tumorigenicity, as evidenced by a pancreatic cancer model in which treatment with TRAIL promoted metastasis formation of xenografted TRAIL-resistant pancreatic cancer cells [80].

### 4.2. Triggering TRAIL/TRAIL-Receptor Signaling for Cancer Therapy

TRAIL/TRAIL-receptor agonists have shown prominent tumoricidal activity in a host of preclinical studies and in a variety of tumor types, whereas no or limited activity was detected towards normal human cells [81]. Based on this promising activity profile, a recombinant trimeric form of TRAIL, named dulanermin, entered phase I/II clinical trials with no apparent toxicity (reviewed in [82]). Dulanermin is further being explored in a multicenter clinical trail, with preliminary results indicating a number of partial responses and stable disease at higher doses of 8 mg/kg [81, 83]. In addition, combination of dulanermin with antiCD20 antibody rituximab yielded 2 complete and 1 partial response in non-Hodgkin lymphoma patients [83, 84], which corresponds to the reported preclinical synergic activity of rituximab and TRAILR agonists [85, 86]. Unfortunately, a recent phase II trial in non small cell lung cancer patients revealed no added benefit of combined Dulanermin and chemotherapy treatment [87].

At the same time, a number of TRAIL-R1 and TRAIL-R2 specific agonistic antibodies have entered clinical evaluation, typically with low to absent toxicity but also without strong clinical benefit as single agent (reviewed in [88]). Thus, clinical experience with both TRAIL and agonistic TRAILR antibodies indicate that real clinical benefit will most likely require combinatorial therapy. Most ongoing clinical trials indeed evaluate combination therapy of TRAILR agonists with, for example, chemotherapy or targeted therapeutics such as the CD20-antibody rituximab as referred to earlier. Such combinatorial approaches may also help to overcome the potential issue of intrinsic resistance to TRAIL, as for instance observed in ~50% of tumor cell lines *in vitro*. In addition, combinatorial treatment strategies may prevent the development of acquired resistance to TRAILR agonists. The potential for acquiring resistance to TRAILR agonists was demonstrated preclinically upon treatment of cancer cells with suboptimal concentrations of an antiTRAIL-R2 antibody. Subsequent repeat treatment with therapeutic concentrations of the same antibody proved ineffective, whereas TRAIL and antiTRAIL-R1 still triggered cell death [89]. From the above it is clear that clinical implementation of TRAILR agonists will most likely be in the context of combinatorial strategies that are specifically designed to trigger synergistic tumoricidal activity.

### 4.3. Targeted TRAIL-Based Cancer Immunotherapy

**Figure 4:**
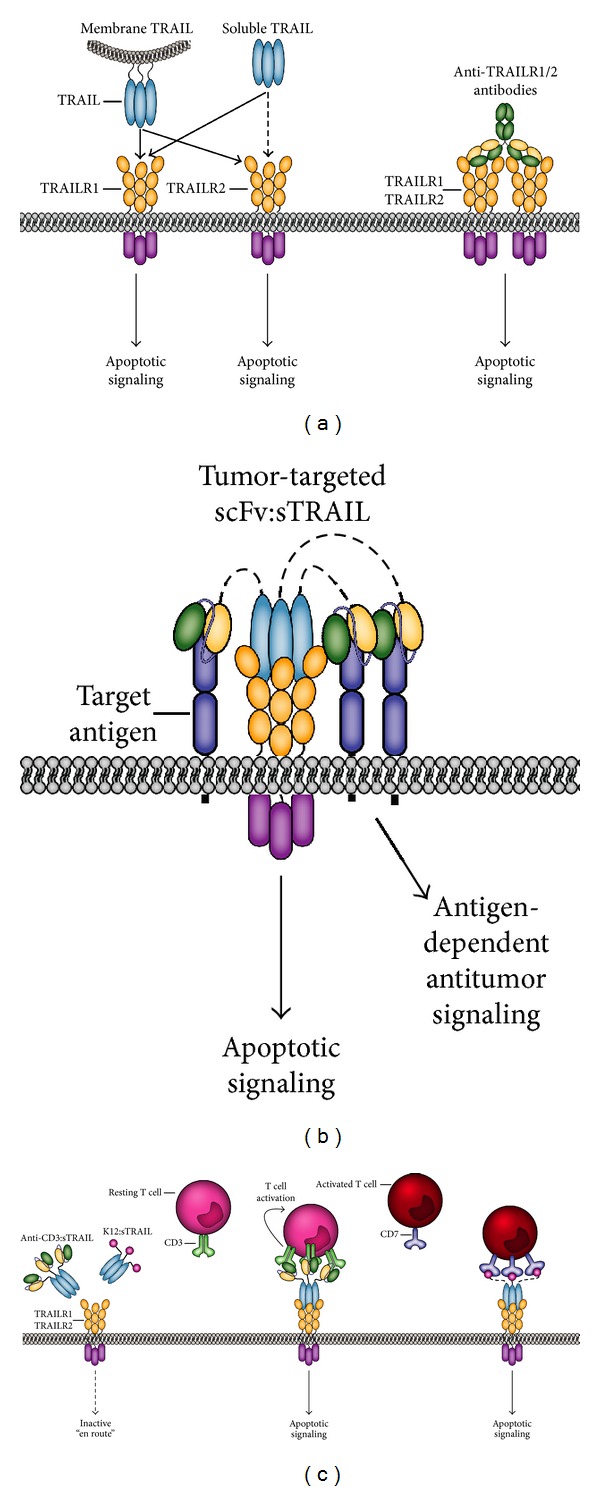
TRAIL/TRAIL-receptor signaling and design of TRAILR agonists. (a) Membrane expressed TRAIL triggers apoptotic signaling via TRAIL-R1 and TRAIL-R2, whereas soluble TRAIL only efficiently activates TRAIL-R1. Recombinant nontargeted sTRAIL thus predominantly triggers TRAIL-R1 apoptotic signaling. Recombinant TRAIL-R1 or TRAIL-R2 agonistic antibodies can selectively activate TRAIL-R1 or TRAIL-R2, respectively. (b) Tumor-targeted delivery of sTRAIL, using scFv:sTRAIL, results in conversion of sTRAIL to membrane-like TRAIL that can induce apoptosis via TRAIL-R1 and TRAIL-R2. The antibody fragment may inhibit or activate target antigen signaling and thereby contribute to the antitumor activity of scFv:sTRAIL. (c) Targeting of T-cell markers CD7 or CD3 with K12:TRAIL and antiCD3:TRAIL, respectively, equips T-cells with membrane-like proapoptotic TRAIL that enhances antitumor T-cell activity. The antiCD3 scFv can also trigger stimulatory signaling in resting T-cells and trigger granzyme/perforin-mediated cytotoxicity.

The signaling characteristics of the TRAIL/TRAIL-receptor system are such that sTRAIL appears well suited for use in antibody fragment-targeted therapy. In particular, whereas sTRAIL has retained receptor activating potential for TRAIL-R1, it cannot efficiently activate TRAIL-R2 signaling (Figure 4(a)) [90]. Indeed, TRAIL-R2 activation by TRAIL is reminiscent of Fas activation by sFasL in that it requires oligomerization to be effectuated. Further TRAIL receptors are ubiquitously expressed in the human body, which will limit tumor accretion of sTRAIL. Tumor accretion will further be limited by the very short half-life of sTRAIL ~30 min in cynamolgus monkeys, a value which closely corresponded to the half-life identified in patients in a phase I clinical trial [81, 91]. 

Fusing sTRAIL to a targeting domain, such as a scFv antibody fragment, will yield a fusion protein with various advantageous properties (Figure 4(b)). First, by virtue of the homotrimerization of sTRAIL, a scFv:TRAIL fusion protein will have an approximate molecular weight of 180 KDa, which is far over the kidney exclusion limit. Consequently, scFv:TRAIL should have a longer circulation half-life. Second, the antibody targeting domain will ensure enhanced tumor-accretion and retention [92–96]. Third, target-selective binding will convert the sTRAIL domain into a membrane-like form of TRAIL that can trigger apoptotic TRAIL-R1 but also TRAIL-R2 apoptotic signaling [97, 98]. The feasibility of this approach has been shown *in vitro* and *in vivo* for both solid tumors and leukemia [92–96]. Of note, scFv:TRAIL binding triggers TRAIL-receptor apoptotic signaling in a monocellular, as well as bi- or multicellular manner. Consequently, neighboring tumor cells that lack the target antigen can also be efficiently eliminated by the so-called bystander effect [99].

Rational choice of tumor target antigen will help optimize the efficacy of scFv:TRAIL-based therapeutics, as evidenced by a study using an EGFR-blocking antibody fragment. Here, the treatment of EGFR-positive tumor cells with scFv:TRAIL inhibited EGFR-mitogenic signaling and simultaneously induced TRAIL-apoptotic signaling [94]. In an analogous fashion, targeting of the melanoma antigen MCSP (Melanoma-Associated Chondroitin Proteoglycan Sulphate) with an scFv:TRAIL fusion protein triggered dual MCSP-inhibitory signaling and TRAIL apoptotic signaling [100]. MCSP is implicated in metastatic behavior of melanoma cells [101, 102]. Interestingly, this fusion protein was >100-fold more effective in colony formation assays that assess metastatic potential than in direct apoptotic assays. Furthermore, TRAIL inhibition only partly blocked the tumoricidal effect in colony formation assays, pointing to MCSP-related therapeutic effects. Thus, scFv-mediated tumoricidal activity can markedly contribute to the therapeutic activity of scFv:TRAIL fusion proteins.

In addition to direct tumor-targeting, the scFv targeting domain can also be used to selectively deliver TRAIL to the cell surface of immune effector cells, whereby these cells are equipped with an additional tumoricidal effector molecule (Figure 4(c)). In a proof of concept study, such T-cell targeted delivery of TRAIL, to the T-cell surface antigen CD3 or CD7, potentiated *in vitro* antitumor T-cell activity ~500-fold and triggered potent tumoricidal activity in an *in vivo* engraftment model [103]. Of note, targeted delivery to CD3 on T-cells also triggered T-cell activation and potentiated intrinsic cytolytic granzyme/perforin pathway signaling. From this study it is apparent that rational choice of T-cell target antigen may be used to generate dual function scFv:TRAIL fusion proteins that on the one hand costimulate T-cells and on the other hand provide the TRAIL-apoptotic signal for tumor cell killing.

### 4.4. Perspectives for Use of TRAIL in Cancer Immunotherapy

From the above it is clear that nontargeted homotrimeric sTRAIL is safe, but also has sub-optimal apoptotic activity. Indeed, it is clear that design of combinatorial strategies of TRAIL with chemotherapy or other targeted drugs is warranted to achieve clinical efficacy. In preclinical studies, TRAIL activity is synergized by a plethora of different established and experimental anticancer drugs. A guideline for such clinical combinatorial TRAIL-based therapy may be the choice for a therapeutic that will block potential TRAIL-mediated prometastatic signaling via NF*κ*B activation, as reported in preclinical studies for TRAIL-resistant pancreatic cancer cells [80]. NF*κ*B activation can be effectively blocked by clinically available proteasome inhibitors such as bortezomib [104]. For such combinatorial strategies, a lack of hepatocyte toxicity is particularly important, as aggregated forms of sTRAIL strongly reduce hepatocyte viability *in vitro* [105] and proteasome inhibition was shown to sensitize hepatocytes to sTRAIL [106]. Nevertheless, in this latter study hepatoma cells were significantly more sensitive and were eliminated at >40-fold lower bortezomib concentrations than normal hepatocytes, clearly highlighting a therapeutic window for this combination.

Inclusion of sTRAIL into an scFv:TRAIL fusion protein can be used to optimize both tumor-selective accretion and apoptotic activity, which may further be enhanced by rational incorporation of a tumoricidal scFv. As for non-targeted TRAIL, combinatorial strategies may help to optimize activity. In this respect, a recent report identified promising synergy between bortezomib and an EGFR-targeted scFv:scTRAIL fusion protein towards hepatoma cells in the absence of toxic hepatocyte activity [107]. Further, depending on the relative contribution of the agonistic TRAIL receptors within a type of tumor, engineered sTRAIL variants that selectively trigger TRAIL-R1 or TRAIL-R2 may be used to enhance tumoricidal activity. For instance, the use of a designed TRAIL mutant with enhanced selectivity for TRAIL-R1 proved significantly more potent in ~50% of EGFR-positive carcinoma cell lines analyzed [92]. Thus, contributory target antigen signaling as well as the tumor type intrinsic characteristics of TRAIL-R1/TRAIL-R2 signaling should be taken into account to identify the optimal scFv:TRAIL protein for respective tumor types.

Additional approaches to optimize TRAIL-based therapy include the engineering of a single chain TRAIL (scTRAIL) protein, in which three TRAIL monomers have been genetically linked [108]. This scTRAIL is a stable homotrimer and has been incorporated into an scFv:scTRAIL format that targets Her-2, with prominent Her-2 restricted *in vitro* and *in vivo* activity [108]. Of note, further genetic engineering of an EGFR-targeted scFv:scTRAIL yielded a dimerized EGFR-targeted scFv:scTRAIL (Db-scTRAIL) that proved to have 5–10 fold higher EGFR-restricted activity than the corresponding trimer form [109].

## 5. Costimulatory TNF Ligands as Inducers of Effective Antitumor Immunity

Development of the adaptive T-cell immune response is highly regulated and depends on an elaborate interplay between T-cell mediated recognition of antigens in the context of major histocompatibility complexes and the prevailing balance of co-stimulatory and immune-inhibitory signals. Unfortunately, this intricate multistep system of T-cell activation provides tumor cells with ample opportunity to interrupt this process. Indeed, tumor cells have been shown to hijack components of the T-cell co-stimulatory system and turn it against the infiltrating T-cell by downregulation of co-stimulatory and up-regulation of immune inhibitory signals [110]. In this situation, T-cell receptor interaction with peptide-loaded major histocompatibility complexes results in T-cell apoptosis or anergy and a halted tumor immune response. Further, many tumors have an altered T-cell balance, with, for example, an increased number of regulatory T-cells that serve to limit effector T-cell responses [111]. Overcoming these tumor-imposed brakes on immunity can help to reestablish antitumor immune responses. This is perhaps best evidenced by the strong clinical benefit of therapeutic antibodies against immune inhibitory signals, with the recent FDA approval of the antiCTLA4 antibody ipilimumab as well as the prominent clinical responses with antiPD-1 and PD-L1 antibodies reported in clinical trials with advanced stage cancer patients [112–114].

In addition, insufficient immune co-stimulatory signaling by tumor necrosis factor (TNF) and TNF-receptor (TNFR) superfamily members further limits the induction of effective antitumor T-cell immunity. Indeed, co-stimulatory TNFR signaling is pivotal for effective T-cell immunity and is also pursued as a therapeutic strategy to induce or restore effective antitumor immunity [7, 88]. Proof of principle for the validity of this approach was first obtained over 2 decades ago using the TNF ligand Lymphotoxin- (LT) alpha. LT-alpha is a soluble ligand that binds to TNFR1 and TNFR2, but like sTNF only effectively stimulates TNFR1 [115, 116]. Targeted delivery of LT-alpha induced *de novo* formation of lymphoid-like tissue in the tumor microenvironment, leading to T-cell mediated antitumor rejection. Unfortunately, LT-alpha also signals via TNFR1, with the potential for systemic effects as found for sTNF, which precludes its systemic administration to humans.

**Figure 5:**
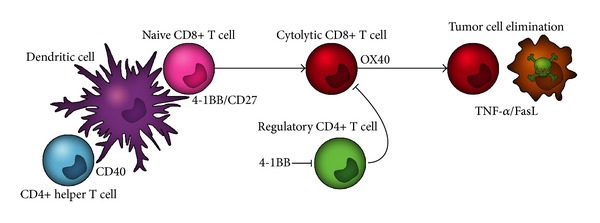
Co-stimulatory TNF ligands provide crucial signals for generation of antitumor T-cell responses. Within the antitumor T-cell immune response, CD40L-mediated CD40 costimulation of DCs by CD4+ T-helper cells is critical for the generation of CD8+ T-cell responses. 4-1BBL expressed on DCs stimulates the generation of T-cell response, while at the same time inhibiting the formation of inducible regulatory T-cells in the tumor micro-environment. CD70 serves to efficiently prime CD4+ and CD8+ T cell responses, to enhance T-cell survival, and to optimize effector function. OX40 is transiently upregulated upon T-cell activation and enhances clonal expansion, survival, proinflammatory cytokine production, and generation of memory CD4 T-cells and enhances CD8+ T-cell survival and expansion.

In the past decades several co-stimulatory TNFL/TNFR pairs have gained prominence as possible therapeutic modulators of T-cell immunity, in particular CD40L/CD40, CD70/CD27, 4-1BBL/4-1BB, and OX40L/OX40 (Figure 5). These various TNF ligands provide a co-stimulatory signal at distinct stages of the immune response to ensure the ultimate generation of functional immunity [117]. A host of preclinical data indicated that reactivation of antitumor T-cell immune responses using agonistic antibodies that target respective co-stimulatory receptors triggers potent antitumor immunity in mouse studies. 

However, an important concern with the use of agonistic antibodies that target the co-stimulatory TNFL/TNFR axis is the potential for deleterious side-effects due to ubiquitous TNFR signaling. Indeed, clinical trials with 4-1BB agonistic antibodies have been terminated after severe hepatic adverse events [118], while clinical trials with CD40 agonists were also associated with significant toxicity [119, 120]. Thus, it is of great interest to design strategies that restrict co-stimulation and subsequent immune activation to the cancer lesion. In the section next I will discuss the biology of CD40L/CD40, CD70/CD27, 4-1BBL/4-1BB and OX40L/OX40 and highlight their current status for cancer immunotherapy. Further, I will discuss the rationale for incorporating these ligands into antibody-fragment targeted delivery of soluble TNFLs to the tumor. 

## 6. CD40L Biology

CD40L is a type II transmembrane protein transiently expressed on activated CD4+, CD8+, and *γδ* T-cells [121]. Further, CD40L expression has been identified on monocytes, activated B-cells, epithelial cells, endothelial cells, platelets, smooth muscle cells, and DCs. Expression of its cognate receptor CD40 is found on B-cells, monocytes, macrophages, platelets, DCs, eosinophils, and activated CD8+ T-cells [122–124]. 

One of the main functions of the CD40L/CD40 system is to activate and “license” DCs to prime effective cytotoxic CD8+ T-cell responses [125, 126]. In brief, co-stimulatory CD40L on antigen-specific CD4+ T helper cells interacts with CD40 on DCs, which triggers a multipronged response with upregulation of CD40, increased DC survival, upregulation of T-cell co-stimulatory molecules CD80 and CD86, an increase in the expression and stability of MHC class II-peptide complexes, and induction of inflammatory cytokines, such as immunostimulatory cytokine IL-12 [127, 128]. Together, these effects serve to “license” DCs and to stimulate the generation of effective CD8+ T-cell response.

In the absence of CD40 signaling, activation of CTLs by so-called “unlicensed” DCs induces T-cell anergy or T-cell deletion and generates regulatory T cells [129]. Importantly, induction of CD40 signaling on DCs using, for example, agonistic antibodies can substitute for CD4+ T-cell help and directly stimulate a specific CD8+ CTL response [125, 130], highlighting a clear rationale for CD40-based cancer immunotherapy.

### 6.1. Triggering CD40L/CD40 Signaling for Cancer Therapy

**Figure 6:**
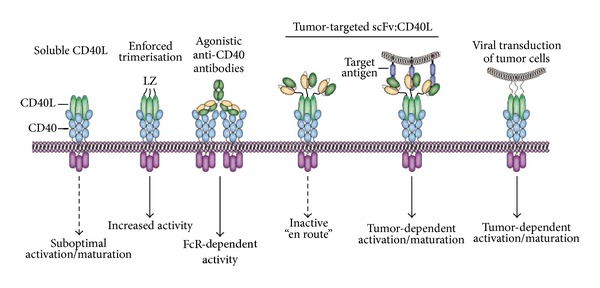
CD40L/CD40-based agonists for cancer therapy. Soluble CD40L is only capable of sub-optimal CD40 signaling. However, enforced trimerization of CD40L or agonistic antiCD40 antibodies can trigger effective CD40-signaling, but with severe side-effects due to ubiquitous CD40-activation. Of note, CD40 agonist antibodies require FcR-mediated cross-linking for effective CD40-signaling. In an antibody fragment-targeted scFv:CD40L fusion protein, the CD40L domain is relatively inactive en route, but gains membrane-like activity upon target antigen-mediated binding. Further, CD40L can be virally transduced into tumor cells, using AdCD40L, to optimize CD40-mediated co-stimulation.

The major aim of therapeutic targeting of CD40 is the induction of efficient DC-mediated priming of T-cell immunity and ensuing induction of effective antitumor T-cell immune responses. This aim has been pursued using various approaches, first and foremost with agonistic antibodies and recombinant forms of soluble CD40L (Figure 6). A human CD40L variant fused to an isoleucine zipper trimerization domain yielded prominent induction of T-cell immunity and tumoricidal activity in preclinical models (Figure 6) [131]. Similarly, agonistic CD40 antibodies yielded potent antitumor T-cell immunity in mice [132]. Both recombinant CD40L and an agonistic antiCD40 antibody (CP-870.893) advanced into early stage clinical trials in cancer patients [119, 120]. In these clinical trials, systemic treatment was associated with significant and dose-limiting toxicity, with the MTD of sCD40L already reached at the low dose of 0.1 mg/kg. Nevertheless, CP-870.893 induced partial responses in 15%–20% of advanced stage melanoma and pancreatic adenocarcinoma patients [133–135], highlighting the promise of CD40 targeting.

Of note, CD40 is also expressed on various malignancies [136]. On many of these malignant cells, the cross-linking of CD40 triggers apoptotic cell death or inhibits proliferation, as for instance, seen upon CD40L treatment of primary ascites-derived ovarian carcinoma cells [137]. This contrasts with the typical CD40-mediated prosurvival and proliferative signaling on normal human cells. CD40-mediated inhibition of proliferation has also been observed in high grade B-cell lymphoma derived cell lines *in vitro* as well as *in vivo*, suggesting that cytotoxic antiCD40 therapy is a potential strategy for B-cell lymphoma. In line with this, the antiCD40 antibodies dacetuzumab (SGN-40) and lucatumumab (HCD122) are being evaluated in various phase I/II trials [88]. Thus CD40 targeted therapy may have a dual beneficial effect comprising direct tumor cell signaling, leading to growth arrest or even apoptosis, and DC-dependent stimulatory activity on antitumor T-cell immunity.

### 6.2. Targeted CD40L/CD40-Based Cancer Immunotherapy

Despite the obvious promise of CD40-targeted immunotherapeutics, the applicability is hampered by systemic and dose-limiting toxicity towards normal cells in patients. Indeed, agonist antiCD40 treatment of mice was already associated with considerable inflammatory side effects. Thus, to further advance CD40 as a therapeutic target it is imperative to enhance the selectivity of CD40 stimulation. Accumulating evidence indicates that CD40 signaling is only initiated when CD40 is clustered within the membrane of target cells. In fact, CD40 signaling induced by antiCD40 antibodies critically depends on Fc-receptor positive cells that provide requisite Fc-mediated clustering of CD40 [138]. Thus, in the absence of sufficient cross-linking the antiCD40 signal is ineffective in generating immunostimulatory signals (Figure 6). For soluble CD40L (sCD40L), evaluation of cross-linking requirements revealed that a trimeric Flag-tagged form of sCD40L could already trigger CD40-signaling [139]. Nevertheless, the efficacy of signaling was increased ~10-fold upon antiFlag antibody-mediated cross-linking. In line with this finding, a hexameric form of sCD40L proved fully capable of activating DCs and inducing T-cell responses [139, 140]. Analogously, an oligomeric surfactant protein D (SP-D) CD40L fusion protein efficiently triggered CD40-dependent B-cell proliferative effects, whereas trimeric sCD40L proved minimally active [141]. This oligomeric form of CD40L was recently found to also effectively stimulate antiHIV immune responses in combination with an HIV-1 Gag vaccine [142]. 

Based on these cross-linking requirements for CD40/C40L signaling, CD40L has also been included in a FAP-targeted scFv:CD40L antibody fragment-based fusion protein (Figure 6) [139]. In brief, antibody fragment-specific delivery will ensure FAP-specific accretion and the subsequent display of multimeric/oligomerized CD40L for effective induction of CD40 signaling on DCs. Indeed, FAP-specific binding of scFv:CD40L triggered an ~25-fold decrease in ED50 for IL-8 production in target cells.

In addition to systemic treatment with CD40 agonists various alternate CD40-based strategies have been investigated in order to optimize DC activity. Of those, adenoviral-based immunostimulatory gene therapy using AdCD40L is of particular interest (Figure 6) [143]. In brief, adenoviral infection will trigger Toll-like receptor (TLR) signaling, while the transgene CD40L potentiates DC activity. AdCD40L can be used for *ex vivo* gene modification of tumor cell vaccines or for direct intratumoral injection and has proven efficacious in murine models. Further, AdCD40L proved safe in humans and induced clinical responses in early clinical trials [144–146]. 

In an alternative approach to optimize DC vaccination a bi-specific co-stimulatory diabody comprising antiCD40 and antiCD28 was constructed [147]. This diabody was designed to simultaneously target and activate stimulatory CD40-signaling on DCs and CD28-signaling on naïve T cells. Using this diabody, the strength and duration of T cell/AML-DC interactions and the responsiveness of T-cells to AML antigens was increased. In addition, CD40L can be genetically fused to a vaccine antigen in order to enhance the immunogenicity of the vaccine. For instance, transfection of DCs with a plasmid containing a genetic fusion between the Hepatitis B virus (HBV) S gene and CD40L upregulated DC maturation markers, triggered IL-12 secretion, and stimulated allogeneic T-cell proliferation [148]. Finally, the previously described multimeric SP-D-CD40L fusion protein was recently using in an adoptive T-cell expansion protocol, in which it efficiently expanded and generated APC-like B-cells *ex vivo* that could trigger CD8+ T-cell expansion [149, 150].

### 6.3. Perspectives for sCD40L/CD40 in Cancer Immunotherapy

Agonistic triggering of CD40 holds considerable promise as an anticancer therapeutic strategy and likely will trigger both direct antiproliferative and/or proapoptotic signaling in CD40-positive tumor cells as well as induction of antitumor T-cell immune responses. A main current focus of CD40 agonist research is on the design and evaluation of combined therapy of CD40 agonists with, for example, chemotherapy or other immunomodulators such as antiCTLA-4 antibody. 

For the clinical use of agonistic CD40 therapeutics it is imperative to achieve higher tumor selectivity to ensure an acceptable toxicity profile. Of interest in this respect is a recent report in which antiCD40 antibody was given at low-dose intra or peritumorally in a slow-release formulation [151]. This strategy resulted in local activation of tumor-specific CD8+ T cells without causing systemic toxicity by nonspecific and ubiquitous CTL activation. Enhanced tumor selectively can also be achieved by antibody-targeting strategies such as the scFv:CD40L fusion protein referred to earlier [139]. Further, the requirement of CD40 agonistic antibodies for FcR-mediated cellular cross-linking opens the possibility of generating bi-specific antibodies that on the one hand target a tumor associated antigen and on the other hand comprise an antiCD40 antibody fragment. In principal, CD40 signaling will only be initiated upon tumor specific binding of the bi-specific antibody fragment thus ensuring a restricted CD40 co-stimulatory signaling.

Potential advances on DC-targeted vaccination strategies include CD40L delivery to tumor cells using an oncolytic adenoviral vector [152, 153]. Infection of tumor cells with oncolytic vectors will trigger tumor cell death, thereby boosting tumor antigen release in the tumor micromilieu, while the CD40L transgene is designed to ensure optimal DC/T-cell interaction [152, 153].

Of note, although it is clear that CD40/CD40L signaling can potently augment antitumor immune responses, recent studies also highlight a potential immunosuppressive effect of soluble trimeric CD40L [154]. The serum of cancer patients contains significantly elevated levels of sCD40L compared with healthy donors [155]. Further, recombinant trimeric sCD40L triggered enrichment of myeloid-derived suppressor cells (MDSCs), expansion of T_reg_, and inhibited effector T-cell proliferation [155]. These findings highlight the importance of evaluating possible unanticipated immune inhibitory effects of sub-optimal CD40 activation by CD40L-based agonists and of the influence of serum sCD40L on therapy outcome. This may ultimately lead to inclusion of serum CD40L as a patient stratification marker.

## 7. CD70/CD27 Biology

The ligand CD70 (also known as CD27L) is a predicted type II homotrimeric transmembrane family member of the TNF family [156, 157]. Unlike most other TNF ligands, CD70 has not been found to contain putative cleavage motifs in the extracellular domain and is thus expected to exist only as a transmembrane protein. The expression of CD70 on normal cells is restricted to activated T- and B-lymphocytes and mature DCs [158]. 

The cognate receptor for CD70 is CD27, a type I transmembrane receptor expressed on naive T cells, mature T-cells, memory B-cells, and NK-cells [158]. Cross-linking of CD27 triggers recruitment of the adaptor proteins TRAF2 and TRAF5 to its cytoplasmic domain, leading to activation of canonical and noncanonical NF*κ*B signaling and c-Jun kinase signaling. Of note, CD27 is expressed as a homodimer on the cell surface [159], suggesting that functional CD70/CD27 interaction occurs at least in a hexameric form. In contrast to CD70, CD27 can be proteolytically processed into a soluble form that may serve as a competitive inhibitor [160]. 

The key biological function of CD70 is to efficiently prime CD4+ and CD8+ T cell responses, to enhance T-cell survival, and to optimize effector function [158, 161–163]. As such, CD70 appears to be a crucial co-stimulatory molecule that is required for the induction of T cell immune responses [158]. In particular, CD27 is present on resting T-cells and is further upregulated upon T-cell activation [156, 164, 165]. This upregulation of CD27 on effector T-cells is transient and strongly correlates with effector function [166]. In line with a critical role for CD27 in T-cell activation, both primary and secondary T cell responses are impaired in CD27^−/−^ mice [167]. CD70/CD27 signaling promotes the development of CD4+ T-cells producing either T_H_1 or T_H_2 type effector cytokines [168], suggesting that CD70/CD27 signaling can trigger a broad spectrum of immune responses. 

### 7.1. CD70/CD27 as Targets for Cancer Immunotherapy

**Figure 7:**
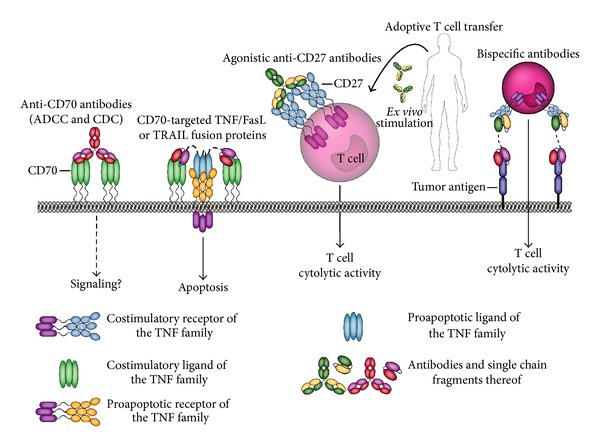
CD70/CD27-based agonists for cancer therapy. CD70 is highly expressed on malignant cells and thus a bona-fide target for antiCD70 antibody based therapy. Similarly, scFv-targeted TRAIL/FasL-based fusion proteins could be used to selectively deliver and locally activate proapoptotic signaling. Triggering CD27 T-cell co-stimulatory signaling may be particularly applicable in, for example, *ex vivo* expansion of adoptive T-cells. Incorporation of an antiCD27 scFv in a bispecific antibody format with a tumor-specific targeting antibody fragment may open up ways to ensure selective modulation and/or inhibition of CD27 signaling in the tumor micro-environment.

Many types of hematological and solid tumors have been documented to express CD70 on the cell surface [169], whereas CD70 is only transiently expressed on antigen-activated lymphocytes on normal cells. This expression pattern establishes CD70 as a potential target for antibody-based cancer therapy (Figure 7). Indeed, antiCD70 antibodies have shown considerable preclinical efficacy towards hematological malignancies. For instance, the humanized IgG1 antibody SGN-70 eliminated CD70-positive tumor cells leading to tumor regression in disseminated lymphoma and multiple myeloma xenograft models [170], in this case via typical antibody effector functions, such as ADCC and CDC. 

In addition to its tumor-restricted expression pattern research has focused on the potential reactivation of antitumor T-cell immunity by agonistic CD27-targeted therapy (Figure 7). The rationale for this approach is emphasized by the more efficient T-cell response in human CD70 transgenic mice upon tumor challenge [161]. Correspondingly, agonistic triggering of CD27-signaling ensured generation of a tumor-specific T-cell response and protected against lymphoma, melanoma, and fibrosarcoma tumor growth upon i.v. or s.c. tumor challenge [161, 171–173].

However, a recent study identified that in established tumor models, CD27 signaling actually promoted tumor growth, with a reduction in T_reg_ apoptosis and production of the T_reg_ survival cytokine IL-2 by CD4+ effector T cells (T_Eff_) [173]. In line with this, the frequency of T_regs_ and the growth of solid tumors was reduced in CD27-deficient mice or in wild-type mice treated with an antagonistic CD27 monoclonal antibody. Indeed, such a CD70-mediated expansion of T_regs_ corroborates with the proposed role of CD70 in tumor immune escape in renal cell carcinoma [174]. Thus, in the tumor micro-environment CD70 may promote tumor growth and immune evasion.

### 7.2. Perspectives for CD70/CD27 in Cancer Immunotherapy

CD70 is highly expressed on various types of cancers and as such is a bona fide target for antiCD70 antibody mediated therapy. Thus, antiCD70 antibodies are well-positioned for, for example, B-cell lymphoma, where preclinical data revealed potent tumoricidal activity [170]. Due to its selective expression profile CD70 may also be a target for design of TRAIL, FasL, or TNF- (prodrug-) based antibody fragment-targeted fusion proteins as well as antibody-drug conjugates/immunotoxins (Figure 7). Further advances in CD70 cytotoxic therapy can be anticipated for combination with, for example, cytotoxic debulking therapy.

However, caution is required in terms of exploiting the immunoregulatory role of CD70/CD27 for cancer immunotherapy. Although the CD70/CD27 axis has prominent immunostimulatory activity in *de novo* induced immune responses, its role in the established tumor micro-environment appears paradoxically opposite, with the expansion and prolonged survival of T_regs_ [173]. Thus, it is imperative to consider the aim of CD70/CD27-based immunotherapy and adapt the strategy accordingly. For instance, upon inclusion in, for example, tumor vaccination strategies, where a *de novo* antitumor T-cell response is induced, adjuvant use of a CD27 agonist may help optimize T-cell responses. Similarly, *ex vivo* expansion of T-cells for subsequent adoptive T-cell transfer experiments may benefit from CD27 agonists (Figure 7). In contrast, in a combination approach of CD70/CD27 targeting with conventional cytotoxic therapy, it may be more appropriate to incorporate an antagonistic CD27 antibody. Hereby, the T_reg_-induced brake on existing T-cell immunity is released. Of note, incorporation of CD27 in a bispecific antibody format with a tumor-specific targeting antibody fragment may open up ways to ensure selective modulation and/or inhibition of CD27 signaling in the tumor micro-environment (Figure 7). Further preclinical studies are needed to evaluate whether this dualistic and context-dependent use of CD70/CD27 for cancer therapy is feasible.

## 8. 4-1BBL/4-1BB Biology

4-1BB (also known as CD137) is an inducible co-stimulatory receptor expressed on activated T-cells as well as activated NK-cells but is constitutively expressed on regulatory T-cells [175]. In fact, 4-1BB expression has been used in a recent study as a marker to identify and isolate natural T_reg_ from peripheral blood mononuclear cells [176]. The ligand for 4-1BB, 4-1BBL, is predominantly expressed on activated antigen presenting cells such as Dendritic cells, B-cells and macrophages [175]. Interestingly, DCs not only express 4-1BBL, but can also express 4-1BB upon activation although the consequence of this co-expression is unclear. Co-stimulatory signaling by 4-1BBL/4-1BB proceeds via recruitment of TRAFs to the cytoplasmic domain of 4-1BB [177], which triggers downstream activation of NF*κ*B, PKB, and PI3K pathways and upregulates antiapoptotic proteins such as Bcl-xL [178, 179].

The inducible expression of 4-1BBL on APCs and 4-1BB on T-cells implied an important role for this ligand/receptor pair in T-cell co-stimulation. Indeed, 4-1BB triggering on T-cells using either an agonistic anti4-1BB antibody or recombinant 4-1BBL enhanced the proliferation as well as the cytokine secretion *in vitro* in both CD4+ and CD8+ T-cells [180, 181]. However, 4-1BB co-stimulatory activity appears to preferentially expand CD8+ T cells over CD4+ T cells [182], with enhanced CD8 T cell survival and inhibition of activation-induced cell death [179, 183–185]. In line with this, 4-1BBL^−/−^ mice are characterized by a decreased CD8-specific T-cell response [186–188].

### 8.1. Triggering 4-1BB/4-1BBL Signaling for Cancer Therapy

**Figure 8:**
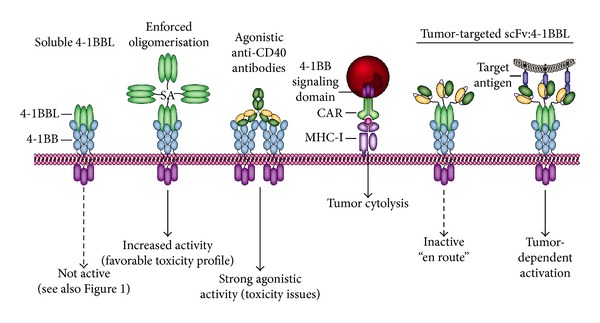
4-1BBL/4-1BB. Soluble 4-1BBL or hexameric 4-1BBL is essentially inactive. However, enforced oligomerization using SA-4-1BBL enables 4-1BB activation with a favorable toxicity profile. In contrast, agonistic 4-1BB antibodies potently activate 4-1BB signaling but with dose-limiting toxicity. The selective use of 4-1BB co-stimulatory signaling can potentiate CAR T-cell activity and trigger effective lysis. Further, selective delivery of 4-1BBL using scFv:4-1BBL ensures target antigen-restricted conversion of inactive s4-1BBL into membrane-like and signaling competent 4-1BBL.

The 4-1BBL/4-1BB co-stimulatory axis has a multifold effect on cancer immunology and has therefore been a prime target for therapeutic manipulation, also based on the finding that various tumor infiltrating T-cells express 4-1BB [189]. Agonistic 4-1BB antibodies trigger effective antitumor immune responses in a variety of mouse models (Figure 8) [190, 191]. However, ubiquitous activation of co-stimulatory 4-1BB signaling was associated with severe toxicity in murine models [192, 193]. Indeed, although in an initial phase I trial the anti4-1BB antibody (BMS-663513) had tolerable side effects, a follow-up Phase II trial revealed severe liver toxicity in ~10% of the patients, with 2 fatalities at doses >1 mg/kg. As a consequence, trials with systemic agonistic anti4-1BB antibody were terminated, although a dose-escalation study has resumed (NCT01471210). These data point to the fact that more tumor-selective 4-1BB activation is needed. In this respect, a recombinant fusion protein comprising streptavidin (SA) fused to murine soluble 4-1BBL, yielding oligomeric 4-1BBL proved safe and effective in murine models (Figure 8) [194]. SA-4-1BBL was also reported to inhibit the formation of induced T_reg_ (iT_reg_), that normally limit T-cell immune response in the tumor micro-environment, and to make CD4+ and CD8+ effector T-cells (T_Eff_) cells refractory to T_reg_ activity [195]. Consequently, the intratumoral T_Eff_/T_reg_ cell ratio increased, which correlated with therapeutic efficacy in various preclinical tumor models. Such an elevated T_Eff_/T_reg_ ratio is predictive of survival in various types of cancer, for example, in ovarian cancer patients [196]. 

Of note, SA-4-1BBL has also been positioned for vaccination strategies, in which biotinylated primary isolated tumor cells can be rapidly loaded with 4-1BBL [197]. Here, 4-1BBL provided co-stimulation and optimized vaccine-mediated T-cell immune responses. The efficacy of DC-based vaccines can also be enhanced by gene-pulsing DCs *ex vivo* with 4-1BBL [198]. Such 4-1BBL expressing DCs trigger enhanced T-cell activation and increased IFN-*γ* production, suggesting that 4-1BBL is a suitable adjuvant to optimize DC-based cancer immunotherapy. 

4-1BB signaling has also been exploited to enhance the efficacy of so-called Chimeric Antigen Receptor (CAR) T-cells. In brief, a CAR is a T-cell transduced with a modified T-cell receptor that comprises the intracellular signaling domain of the CD3 zeta chain fused to an extracellular antitumor scFv antibody fragment [199]. This antitumor antibody fragment retargets the CAR to tumor cells, whereupon the CD3 zeta chain triggers T-cell activation. More recent CAR T-cells have been engineered to additionally contain the intracellular signaling domain of 4-1BB, which provides a second co-stimulatory signal (Figure 8). A recent clinical trial with a CD19 specific CAR yielded exciting effects, with the infusion of moderate dose autologous CAR T-cells triggering therapeutic activity in all three advanced CLL patients and a complete remission in 2 of the patients [200, 201]. An important cautionary note for including co-stimulatory domains in CAR T-cells was learned when an Her2-specific CAR containing dual intracellular 4-1BB and CD28 co-stimulatory domains proved fatal upon infusion in the patient [202].

The 4-1BBL/4-1BB co-stimulatory axis has been mainly investigated in terms of T-cell immune responses. However, the antitumor efficacy of 4-1BB stimulation in mice appears to partly rely on NK-cell activity [203]. In line with this, *ex vivo* stimulation of PBMCs of healthy donors or patients with renal cell or ovarian carcinoma with a combination of soluble 4-1BBL and IL-12 induced a long-term proliferation of functional CD56^bright^ NK cells [204]. These data further confirm the role of 4-1BBL/4-1BB in NK-cell biology and highlight that inclusion of s4-1BBL may optimize *ex vivo* expansion and activation of NK cells for cancer immunotherapy. 

### 8.2. Targeted 4-1BBL-Based Cancer Immunotherapy

As with the other ligand/receptor pairs discussed in this review, both 4-1BB and 4-1BBL are naturally occurring homotrimers. However, activation of 4-1BB with 4-1BBL requires oligomerization, with trimeric soluble 4-1BBL being approximately 100-fold less active than oligomerized 4-1BBL [139]. This differential activity profile positions 4-1BBL as a potential effector domain for antibody fragment-targeted immunotherapy. Indeed, proof of concept for restricted activation of 4-1BB-mediated co-stimulation was obtained for targeted delivery of 4-1BBL to the tumor stroma marker FAP using an scFvFAP-1BBL fusion protein (Figure 8) [205]. In cocultures of FAP-expressing HT1080 cells and T-cells, the fusion protein provided co-stimulatory signals. In contrast, in cocultures with parental FAP-negative HT1080 and T-cells, scFvFAP:4-1BBL was inactive. Together, these reports indicate that target cell-dependent co-stimulation with scFv:4-1BBL may enhance tumor-restricted T-cell activation and improve T cell-mediated antitumor immunity.

### 8.3. Perspectives for 4-1BBL/4-1BB in Cancer Immunotherapy

Although systemic 4-1BB treatment is associated with severe toxicity, the 4-1BB/4-1BBl axis remains of great interest and various approaches may be used to increase tumor specificity. For instance, a recent report explored spatiotemporal infusion of adoptive T-cell transfer and 4-1BB. In brief, adoptively transferred T-cells were first infused and were found to upregulate 4-1BB expression only at the site of tumor, thus yielding a therapeutic window for 4-1BB agonist treatment 3 days after lymphocyte infusion [206]. In addition, combined use of 4-1BBL with T-cell retargeting strategies may yield an MHC-unrestricted potent antitumor immune response. The potential for such a strategy has been recently shown, with a combination of T-cell retargeting bispecific antibody and scFv:4-1BBL fusion protein, yielding a strongly enhanced cytotoxic T-cell response [207]. In brief, the bispecific antibody provided MHC-unrestricted tumor recognition and the primary CD3-mediated activation signal, whereas endoglin-targeted 4-1BBL provided the crucial second T-cell activation signal. Further, combination of a 4-1BBL-expressing tumor cell vaccine with antibody-mediated blockade of CTLA-4 proved superior to tumor cell vaccine alone [208]. Here, the combination triggered regression of established tumors and yielded a significant increase in survival in mice. Similarly, tumor targeted delivery using scFv:4-1BBL may be used to maximize CAR T-cell activity. Here, sequential dosing may help to optimize tumor-restricted CAR activation and improve on the safety profile of the CAR technology.

## 9. OX40L/OX40 Biology

Another prominent candidate for the (re)activation of active T-cell immunity is the ligand/receptor pair OX40L/OX40. OX40 (CD134) was named after the antibody clone OX40 that was originally used to identify this receptor on activated CD4+ T-cell blasts [209]. OX40 was later also identified on CD8+ T-cells, NK cells, NKT cells, and neutrophils [210]. OX40L is primarily expressed on antigen presenting cells, such as DCs, B-cells, and macrophages, but is also expressed on freshly isolated NK-cells where it is further upregulated upon activation [211]. In addition, OX40L is expressed on the basal side of endothelial cells at sites of inflammation, where it may provide a crucial co-stimulatory signal to extravasated T-cells. 

OX40 intracellular signaling proceeds in particular via TRAF2 and TRAF5 and activates downstream canonical and noncanonical NF*κ*B signaling, PI3K and PKB pathways. OX40 signaling upregulates the antiapoptotic proteins Bcl2, Bcl-xL, and survivin ~4–6 days following TCR-ligation and thereby provides a crucial survival signal to CD4+ and CD8+ T-cells [212–214]. Indeed, in OX40^−/−^ T-cells the expression of these antiapoptotic proteins is downregulated [212]. OX40 provides a co-stimulatory signal to sub-optimally and low-dose TCR-stimulated T-cells and enhances clonal expansion, survival, proinflammatory cytokine production, and generation of memory CD4 T-cells [215–218]. Further, OX40 also directly enhances CD8+ T-cell survival and expansion as well as indirectly stimulates CD8+ T-cell expansion via induction of CD4+ T-cell helper responses [219, 220].

Importantly, expression of OX40 on T-cells is transient and first becomes detectable ~12–24 h after T-cell receptor (TCR) ligation on both activated CD4+ and CD8+ T-cells [221]. After 48–96 h, the expression of OX40 on these activated T-cells is downregulated [221]. This temporal expression profile of OX40 corroborates with the required timing of therapeutic OX40 antibodies, which need to be given 1-2 days after antigenic stimulation [215]. During antigen recall, OX40 is rapidly expressed on memory T-cells within 1–4 h. Of note, OX40 is also constitutively expressed on T-regulatory (T_reg_) cells in mice [166], but human T_reg_ express no or minimal OX40 although it can be up-regulated during inflammation.

### 9.1. Triggering OX40L/OX40-Signaling for Cancer Therapy

**Figure 9:**
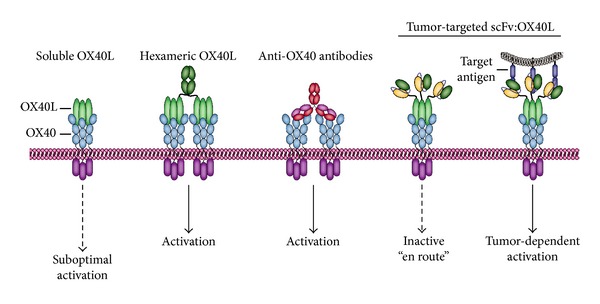
OX40L/OX40. Soluble OX40L can only suboptimally activate OX40 co-stimulatory signaling. Hexameric recombinant OX40L is fully capable of activating OX40-signaling like OX40 agonistic antibodies, with no dose-limiting toxicity of such an OX40 antibody in an early clinical trial. To increase tumor selectivity, sOX40L can be targeted to tumor cells using scFv:OX40L. The sOX40L domain will convert into membrane-like and fully signaling competent OX40L only upon selective binding to the targeted antigen.

Since the initial report describing OX40 expression on tumor-infiltrating T-cells in melanoma and head and neck cancer patients [222], the therapeutic targeting of the OX40L/OX40 axis for cancer therapy has been pursued using agonistic OX40 antibodies or recombinant forms of soluble OX40L (e.g., OX40L:Fc) (Figure 9) [131, 223]. In various preclinical models of immunogenic tumors, such as CT26 colon cancer and MC303 sarcoma, OX40 antibody treatment was shown to eradicate tumor outgrowth [223, 224]. Based on these results a clinical trial was initiated with a murine antiOX40 monoclonal antibody (9B12). In this phase I trial of 30 patients a single bolus injection of 9B12 did not reach the maximum tolerated dose (MTD) and was associated with a relatively mild toxicity profile [225]. Importantly, patients receiving antiOX40 treatment had an increase in tumor-specific immune responses after therapy and had increased CD4+ and CD8+ T-cell proliferation. However, no objective clinical responses were detected, although some patients did experience tumor shrinkage. Of note, the detection of human antimouse antibody (HAMA) responses after single treatment highlights the need for development of a humanized antiOX40 antibody, which is currently being pursued. An alternative OX40 agonist is a recombinant hexameric human OX40L:Fc, comprising a trimerizing isoleucine zipper (ILZ) domain, which had superior biological activity as soluble therapeutic *in vitro* compared to antiOX40 antibody treatment [131], positioning OX40L-based therapeutics as viable alternative to agonistic OX40 antibodies.

However, in poorly immunogenic tumors single agent antiOX40 treatment does not provide adequate antitumor immunity, as a result of which combination of OX40 with other strategies has been pursued. In this respect, combination of OX40 with the T_reg_ depleting chemotherapeutic drug cyclophosphamide triggered a combined depletion of intratumoral regulatory T-cells and an influx of CD8+ CTL, which together yielded strong tumoricidal activity [226]. In line with this report, T_reg_ depletion was also observed in various other preclinical models with OX40 agonists [224, 227]. Synergistic tumoricidal activity was further identified in preclinical studies for combination of OX40 agonists with, for example, cytokines such as IL-2 [228], with tumor vaccination approaches [229], with adoptive T-cell transfer [189], and by combination with a 4-1BB agonist antibody [193].

### 9.2. Targeted OX40L-Based Cancer Immunotherapy

The previously described antibody-based OX40 agonist approaches can potentially activate the OX40L/OX40 ligand/receptor pair in a ubiquitous manner, which may translate into unanticipated off-target effects particularly upon combinatorial OX40-based therapeutic approaches. To achieve more selective tumor-specific activation of OX40 signaling on T-cells, the OX40L can be incorporated into an antibody fragment-targeted approach. Specifically, soluble homotrimeric OX40L does not or only minimally induces OX40 signaling [230]. In contrast, hexameric OX40L proved to be fully capable of activating OX40 [230], a finding corroborated by the previously published co-stimulatory activity of the hexameric OX40L:Fc fusion protein [131, 230]. Based on the previously described characteristics of the OX40/OX40L signaling pathway, OX40L seems particularly amenable to targeted delivery and activation. Indeed, antibody fragment-mediated delivery of sOX40L to the stroma marker FAP revealed potent OX40-signaling activity on targeted cells (Figure 9) [230]. In contrast, on FAP-negative cells this fusion protein remained essentially inactive. Thus, this novel fusion protein appears to fulfill the prerequisite of target-cell restricted activity and may be a useful agent for the selective enhancement of OX40-mediated T-cell co-stimulation in the tumor micro-environment.

## 10. Conclusions

As discussed in this review, both proapoptotic and co-stimulatory TNFL/TNFR ligand/receptor pairs hold considerable promise for immunotherapy of cancer, with various agonistic TNFR antibodies and recombinant soluble TNFLs poised for or undergoing clinical evaluation. Indeed, the host of ongoing preclinical studies and promising early clinical results suggests that targeting of the TNFL/TNFR axis will become part of clinical practice in the near future. 

However, the ubiquitous activation of proapoptotic or co-stimulatory TNFR signaling can have severe side-effects, as evidenced by the early clinical experience with systemic TNF infusion as well as the recent experience with systemic agonistic 4-1BB antibody treatment. Thus, tumor-restricted activation is being pursued in order to fully capitalize on the therapeutic potential of this regulatory axis. In this respect, antibody fragment-based fusion proteins of the various TNFLs discussed here hold considerable promise. Such targeted TNFLs selectively bind to a tumor target antigen and, by virtue of the often reduced activity of soluble homotrimeric ligand, are relatively inactive “en route.” However, once bound to the target antigen the ligand will acquire tumoricidal or co-stimulatory activity resembling that of the corresponding transmembrane ligands. For promiscuously active ligands such as TNF the incorporation into advanced prodrug strategies can further help to ensure strictly tumor-localized unmasking of apoptotic activity. Thus, this targeting approach may make significant headway towards a “magic bullet” with maximum cancer-selective activity and minimal side effects. 

Further, as evident from early clinical trials with TRAIL-receptor as well as CD40 agonists, single agent treatment of patients will likely not yield sufficient clinical benefit. In order to achieve meaningful clinical responses, design of rational combinatorial strategies that ensure maximal synergistic tumoricidal activity and minimal toxicity are called for. Many such approaches have been initiated and are currently being evaluated in preclinical studies as well as early phase clinical trials. Of interest for proapoptotic ligands/receptors are combinations that converge on known antiapoptotic regulators of Death Receptor-mediated apoptosis. For co-stimulatory ligands/receptors, promising strategies include those that shift the immune-inhibitory tumor micro-environment towards an immune stimulatory tumor micro-environment by, for example, targeted depletion of regulatory T-cells and myeloid-derived suppressor cells.

Of note, an important issue that needs to be addressed for any new type of immunotherapy to enter clinical practice is the identification of appropriate patient stratification criteria. In this respect, CD8+ T-cell infiltration and CD8+/T_reg_ ratios have prognostic value for patient survival and may also be used to identify patients likely to respond to immunotherapy [231]. The clinical activity of ipilimumab, for instance, correlated well with high baseline expression of FoxP3 and indoleamine 2,3-dioxygenase (IDO), an immunoregulatory enzyme that suppresses T-cell responses, and high numbers of tumor-infiltrating lymphocytes in a prospective phase II clinical trial and these markers may thus be of use in patient selection [232]. Thus, research in the upcoming years should focus on not only identifying integrated TNFL/TNFR-based combinatorial immunotherapeutics but also on the identification of appropriate patient selection criteria.

## Abbreviations

TNF: Tumor necrosis factor
Fas: Fibroblast-associated cell-surface
FasL: Fibroblast-associated cell-surface ligand
TRAIL: TNF-related apoptosis-inducing ligand
TNFR1: TNF receptor 1
TNFR2: TNF receptor 2
TRAIL-R1: TRAIL receptor 1
TRAIL-R2: TRAIL receptor 2
DD: Death domain
LT*α*: Lymphotoxin alpha
scFv: Single chain fragment of variable regions
NF*κ*B: Nuclear factor kappa B
FADD: Fas-associated death domain
TRADD: TNFR-associated via death domain
cIAP: Cellular inhibitor of apoptosis protein
TRAF2: TNF-receptor associated factor 2
cFLIP: Cellular FADD-like IL-1b-converting enzyme inhibitory protein
FAP: Fibroblast activation protein
DcR3: Decoy receptor 3
DISC: Death inducing signaling complex
EGFR: Epidermal growth factor receptor
HER2: Human epidermal growth factor receptor 2
IL13R*α*2: Interleukin 13 receptor alpha 2
Bcl-2: B-cell lymphoma/leukemia 2 gene
Bcl-xL: B-cell leukemia xL.
